# Molecular Phylogeny of the Cliff Ferns (Woodsiaceae: Polypodiales) with a Proposed Infrageneric Classification

**DOI:** 10.1371/journal.pone.0136318

**Published:** 2015-09-08

**Authors:** Yizhen Shao, Ran Wei, Xianchun Zhang, Qiaoping Xiang

**Affiliations:** 1 State Key Laboratory of Systematic and Evolutionary Botany, Institute of Botany, Chinese Academy of Sciences, Beijing, 100093, China; 2 University of the Chinese Academy of Sciences, Beijing, 100049, China; University of Florida, UNITED STATES

## Abstract

The cliff fern family Woodsiaceae has experienced frequent taxonomic changes at the familial and generic ranks since its establishment. The bulk of its species were placed in *Woodsia*, while *Cheilanthopsis*, *Hymenocystis*, *Physematium*, and *Protowoodsia* are segregates recognized by some authors. Phylogenetic relationships among the genera of Woodsiaceae remain unclear because of the extreme morphological diversity and inadequate taxon sampling in phylogenetic studies to date. In this study, we carry out comprehensive phylogenetic analyses of Woodsiaceae using molecular evidence from four chloroplast DNA markers (*atp*A, *mat*K, *rbc*L and *trn*L–F) and covering over half the currently recognized species. Our results show three main clades in Woodsiaceae corresponding to *Physematium* (clade I), *Cheilanthopsis*–*Protowoodsia* (clade II) and *Woodsia* s.s. (clade III). In the interest of preserving monophyly and taxonomic stability, a broadly defined *Woodsia* including the other segregates is proposed, which is characterized by the distinctive indument and inferior indusia. Therefore, we present a new subgeneric classification of the redefined *Woodsia* based on phylogenetic and ancestral state reconstructions to better reflect the morphological variation, geographic distribution pattern, and evolutionary history of the genus. Our analyses of the cytological character evolution support multiple aneuploidy events that have resulted in the reduction of chromosome base number from 41 to 33, 37, 38, 39 and 40 during the evolutionary history of the cliff ferns.

## Introduction

**Fig 1:**
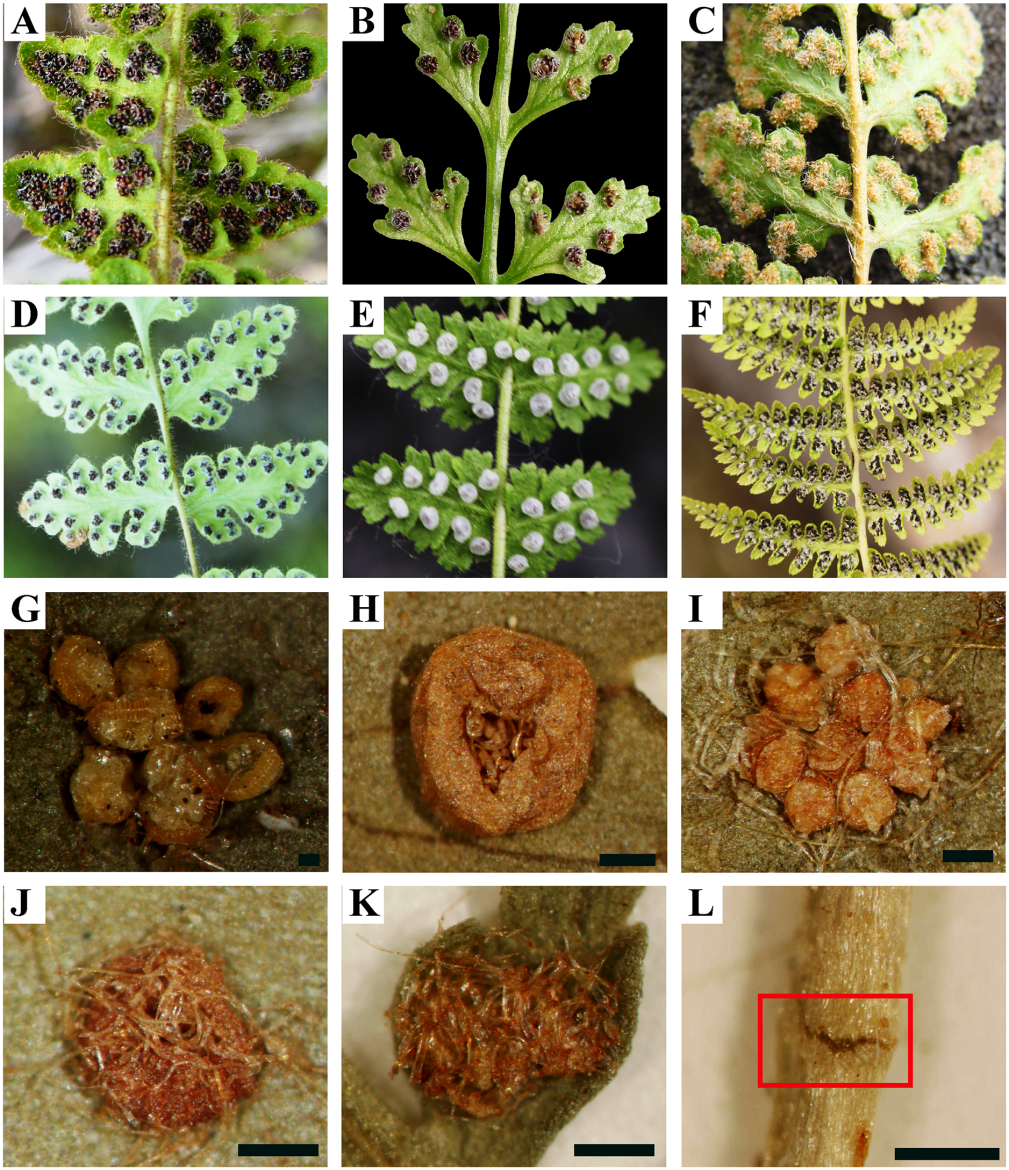
Diversity in leaf shape and indusium morphology in Woodsiaceae. A, *W*. *andersonii*; B, *W*. *glabella*; C, *W*. *ilvensis*; D, *W*. *macrochlaena*; E, *Woodsia* (*Protowoodsia*) *manchuriensis*; F, *W*. (*Cheilanthopsis*) *indusiosa*; G, stem articulation of *W*. *glabella*; H, exindusiate sori of *W*. *cyclobala*; I, globose indusia of *W*. *manchuriensis*; J, curly hairs indusia of *W*. *andersonii*; K, cup-shaped indusia of *W*. *intermedia*; L, saucer-shaped indusia of *W*. *glabella*. Scale bars indicate 500 μm. Photographs: Zhang, X.-C., Shao, Y.-Z.

The cliff fern family Woodsiaceae, as recently circumscribed [1,2], comprises about 35–36 species with its diversity chiefly in boreal or temperate zones of the Northern Hemisphere, and also extending to Central and South America, and southern Africa [3–7]. The cliff ferns are morphologically variable in their leaf shape, indument, and indusia (Fig 1A–1F), and mainly occur on exposed or shaded banks, rock outcrops, or talus slopes.

The delimitation of Woodsiaceae has been contentious since its establishment [1,2,8–13]. Ching [8] defined Woodsiaceae (including *Woodsia* and *Protowoodsia*) based on the inferior indusia that are calyciform, rudimentary-shaped or fringed with long hairs around the edges (Fig 1G–1K). However, some authors placed *Woodsia* s.s. and its allied genera (including *Cheilanthopsis*, *Hymenocystis*, *Physematium* and *Protowoodsia*) into Polypodiaceae [14], Aspidiaceae [15,16], Dennstaedtiaceae [17], Athyriaceae [18], or Dryopteridaceae [19,20], because of superficial morphological similarities in leaf shape, scales, and indument. Smith et al. [12] tentatively defined the Woodsiaceae by including the groups we now recognize as Athyriaceae and Cystopteridaceae [1,2] due to lack of additional information. However, they suggested that denser sampling of these groups would likely shed light on their relationships. The most current molecular phylogenetic studies show support for Woodsiaceae *sensu* Ching [8] as a separate entity from Athyriaceae and Cystopteridaceae [21–28], and this has led to a relatively stable delimitation of Woodsiaceae from closely related families [1,2].

Generic circumscription within Woodsiaceae has been variable as well [1,2,8,10,11,29]. The genera *Physematium* and *Protowoodsia* were segregated from *Woodsia* based on the stem articulations (Fig 1L), the shape of indusia (e.g., globose, filamentous or exindusiate) (Fig 1G–1K), and chromosome base number (*x* = 33, 37, 38, 39, 40, 41) [5,8,10,30], and *Cheilanthopsis* was segregated from *Woodsia* based on the occurrence of false indusia that were similar to *Cheilanthes* (Pteridaceae) [5,10,31]. In a recent taxonomic revision based on morphology, Shmakov [11] maintained the generic level of *Cheilanthopsis*, *Protowoodsia* and *Woodsia* s.s., and resurrected the monotypic genus *Hymenocystis* with one sole representative, *H*. *fragilis*, endemic to the Caucasus. However, Rothfels et al. [2] adopted a single broadly defined genus *Woodsia*, which included *Cheilanthopsis*, *Hymenocystis*, *Protowoodsia* and *Physematium* based on molecular phylogenetic results from multiple plastid markers [28]. Most controversy and uncertainty about generic delimitation in Woodsiaceae is caused by differences in interpretations of morphological diversity. The stem articulation, shape of indusia, indument, and the chromosome base number have often been considered to be taxonomically informative for generic delimitation [3,5,10,19,32]. Unfortunately, many of these features reflect quantitative rather than qualitative variation. Moreover, most of these diagnostic characters and their taxonomic values have not been carefully examined within a phylogenetic framework.

Although several phylogenetic studies have shown the need for re-circumscription within the cliff ferns, species sampling was insufficient to propose an infrafamilial classification. For example, only one species was sampled in Schuettpelz and Pryer [23] and Wang et al. [24], and seven species in Rothfels et al. [28]. In a recent study, Larsson [33] provided a more detailed phylogenetic framework for *Woodsia* with its segregates base on 36 sampled taxa, but this study focused mainly on the evolutionary history of polyploids within the genus.

In this study, we generate a molecular phylogeny for Woodsiaceae based on a more comprehensive taxon sampling than previous studies (e.g., [23,24]). We have three main aims: (1) to test the generic limits, i.e., a broadly defined *Woodsia* versus four or five genera within Woodsiaceae; (2) to investigate the evolution of selected morphological characters that have been traditionally used for infrageneric classification; and (3) to explore the evolutionary pattern of the karyotype. The last question is of particular interest because Ma [32] proposed a hybridization and evolution mechanism of chromosome base number in relation to our understanding of cytological diversity in this family.

## Materials and Methods

### Ethics statement

This study did not require any special permits because all collecting was performed by researchers located at institutes with the permits required such as IBCAS (Institute of Botany, Chinese Academy of Sciences) in Beijing.

### Taxon sampling

Our sampling followed the classification in Wu [6], Windham [4], and Shmakov [11]. A complete list of taxa included in this study is presented in S1 Table, which includes voucher information and GenBank accession numbers. In Woodsiaceae, we sampled 33 specimens representing 21 species, spanning the morphological diversity of *Woodsia*, *Cheilanthopsis*, *Physematium*, and *Protowoodsia*; unfortunately material of the monotypic genus *Hymenocystis* was not available. For each genus we sampled the type species, including *Cheilanthopsis indusiosa* (Christ) Ching [= *Cheilanthopsis straminea* (Brause) Hieron.], *Physematium molle* Kaulf., *Protowoodsia manchuriensis* (Hook.) Ching, and *Woodsia ilvensis* (L.) R. Br. Our sampling covered the distribution range of the family including Eurasia, North America, South America, and southern Africa. In particular, we sampled representatives of most morphological groups within the new world species recognized in Windham [4] and other recent treatments [19,34,35]. For outgroup taxa, we sampled representatives of the families that are considered to be closely related to Woodsiaceae in eupolypods II based on the most recent phylogenetic studies [23,26–28]. Outgroup sampling included Aspleniaceae represented by *Asplenium* and *Hymenasplenium*, Athyriaceae by *Anisocampium*, *Athyrium*, *Cornopteris*, *Deparia*, and *Diplazium*, Blechnaceae by *Blechnum* and *Woodwardia*, Cystopteridaceae by *Cystopteris* and *Gymnocarpium*, Diplaziopsidaceae by *Diplaziopsis*, Onocleaceae by *Matteuccia*, *Onoclea* and *Pentarhizidium*, Rhachidosoraceae by *Rhachidosorus*, and Thelypteridaceae by *Thelypteris*.

### DNA isolation, amplification and sequencing

We extracted genomic DNA using the Tiangen Plant Genomic DNA Kit (Tiangen Biotech Co., Beijing, China). For each taxon, we amplified three chloroplast genes (*atp*A, *mat*K, *rbc*L) and one non-coding region (*trn*L–F) separately with standard polymerase chain reaction (PCR). We amplified the *atp*A region following the primers and procedure of Schuettpelz and Pryer [23]. We amplified the *mat*K region using primers (PolypodF1 and PolypodR1) and PCR protocols introduced by the CBoL Plant Barcoding Working Group (http://www.barcodinglife.org/index.php/Public_Primer_PrimerSearch). We amplified the *rbc*L region using primers 1F and 1351R, following PCR protocol outlined in Hasebe et al. [36] and Murakami et al. [37]. We amplified the *trn*L–F region using the primers and PCR protocol described in Wang et al. [38]. We have listed complete primer information in Table 1. We purified our PCR products using the Tiangel Midi Purification Kit (Tiangen). We conducted sequencing reactions using the DYEnamic ETDye Terminator Cycle Sequencing Kit (Amersham Pharmacia Biotech) and sequenced the products on an ABI 3730XL genetic analyzer (Applied Biosystems, Foster City, USA). We aligned all the sequences obtained using CLUSTAL X [39], and adjusted them manually in BioEdit [40].

**Table 1:** List of primers used for DNA amplification and sequencing.

DNA region	Primer name	Sequence (5′–3′)	Source
*atp*A	ESATPF412F	GARCARGTTCGACAGCAAGT	Schuettpelz et al., 2006
	ESTRNR46F	GTATAGGTTCRARTCCTATTGGACG	Schuettpelz et al., 2006
*mat*K	PolypodF1	ATTTYTGGARGAYAGAYTDCC	CBoL Plant Barcoding Working Group
	PolypodR1	CGTRGTATATATCTCRATYTACGC	CBoL Plant Barcoding Working Group
*rbc*L	1F	ATGTCACCACAAACAGAGACTAAAGC	Hasebe et al., 1994
	1351R	GCAGCAGCTAGTTCCGGGCTCCA	Hasebe et al., 1994
*trn*L–F	c	CGGAATTGGTAGACGCTACG	Wang et al., 2003
	f	ATTTGAACTGGTGACACGAG	Wang et al., 2003

### Phylogenetic analyses

We conducted independent, preliminary maximum likelihood (ML) analyses of each gene region to determine their congruence. We measured congruence among the four chloroplast regions by comparing bootstrap values; bootstrap values less than 75% for conflicting nodes were not considered to be incongruent [41]. Because we found no nodes in conflict, we combined all regions into a single dataset. The methods of preliminary phylogenetic analysis were the same as those described below for the combined dataset.

We analyzed the combined dataset using maximum parsimony (MP), maximum likelihood, and Bayesian inference (BI). We ran the MP analyses in PAUP* v4.0b10 [42], and we weighted all characters equally and treated them as unordered. Heuristic searches with 1000 random sequence-addition replicates were conducted with tree bisection–reconnection (TBR) branch-swapping, and 10 trees from each random sequence addition were saved. We then calculated MP bootstrap support values (BS_MP_) using 1000 replicates with TBR branch swapping, and 10 trees saved per replicate.

For ML and BI analyses, we found the best-fitting model of sequence evolution for each gene using jModelTest v0.11 [43] based on the corrected Akaike information criterion (AICc) [44]. The selected substitution models for each gene are listed in Table 2. ML trees were generated by performing a rapid bootstrap analysis on the RAxML v7.0 [45,46]. After 1000 rapid bootstrap search steps, ML bootstrap values (BS_ML_) of each node were visualized using FigTree v1.4 (available from http://beast.bio.ed.ac.uk/figtree). BI analysis was performed using MrBayes v3.2.1 [47,48]. Three independent Bayesian Markov chain Monte Carlo (BMCMC) runs with one cold and three incrementally heated chains, each for 3,000,000 generations, sampling every 1000 generation, were run starting with a random tree (rates are free to vary across partitions). The convergence of runs, estimation of burn-in, and effective sampling sizes (ESS) were checked using Tracer v1.5 [49]. Bayesian posterior probabilities (PP_BI_) were calculated as 50% majority consensus of all sampled trees after discarding the burn-in phase. The data matrix and phylogenetic trees are available in TreeBase (http://purl.org/phylo/treebase/phylows/study/TB2:S17659).

**Table 2:** Descriptive statistics and selected models based on AICc for analyzed plastid DNA sequence data matrices. AICc, corrected Akaike information criterion; CI, consistency index; RI, retention index.

DNA region	No. of accessions	Aligned length	No. of variable characters (%)	No. of parsimony informative characters (%)	Substitution model	CI	RI
*atp*A	58	1702	591 (34.7)	437 (25.6)	GTR+I+G	0.457	0.726
*mat*K	54	800	537 (67.1)	413 (51.6)	TVM+I+G	0.444	0.732
*rbc*L	61	1206	343 (28.4)	254 (21.1)	SYM+I+G	0.452	0.746
*trn*L−F	53	992	596 (60.1)	426 (42.9)	TVM+G	0.519	0.726
Conbined	61	4700	2070 (44.0)	1533 (32.6)	GTR+I+G	0.462	0.722

### Morphological and cytological character evolution within Woodsiaceae

We investigated two macro-morphological characters, namely stem articulation and shape of indusia [4,6], and chromosome base number, using information obtained from the literature [3,4,6,8,11,32,50,51] and from our own observations of herbarium specimens and in the field.

The evolution of morphological characters was reconstructed using Mesquite v2.7.5 [52]. The missing data were coded as “?” (S2 Table). The data matrix was reduced to a single accession for each species including 29 ingroup and outgroup taxa to represent each family closely related to Woodsiaceae within eupolypod II. To account for phylogenetic uncertainty, characters were plotted onto 1000 input trees that were obtained from the BMCMC analyses of the combined dataset using MrBayes v3.2.1 with the settings: 3,000,000 generations with a sample frequency of one sample every 1000 generations, and a burn-in phase discarding the first 2001 sampled trees. To reconstruct character evolution, a maximum likelihood approach using Markov k-state 1 parameter model (Mk1) [53] was used. Under this model any particular change is equally probable, and the rate of change is the only parameter. The “Trace-characters-over-trees” command was used to calculate ancestral states at each node. The “show-node-absent” option were set to show the proportion of the node with weak support (PP_BI_ < 0.95) across 1000 trees.

### Nomenclature

The electronic version of this article in Portable Document Format (PDF) in a work with an ISSN or ISBN will represent a published work according to the International Code of Nomenclature for algae, fungi, and plants, and hence the new names contained in the electronic publication of a PLOS article are effectively published under that Code from the electronic edition alone, so there is no longer any need to provide printed copies.

In addition, new names contained in this work have been submitted to IPNI, from where they will be made available to the Global Names Index. The IPNI LSIDs can be resolved and the associated information viewed through any standard web browser by appending the LSID contained in this publication to the prefix http://ipni.org/. The online version of this work is archived and available from the following digital repositories: PubMed Central, LOCKSS.

## Results

### Phylogenetic analyses

**Fig 2:**
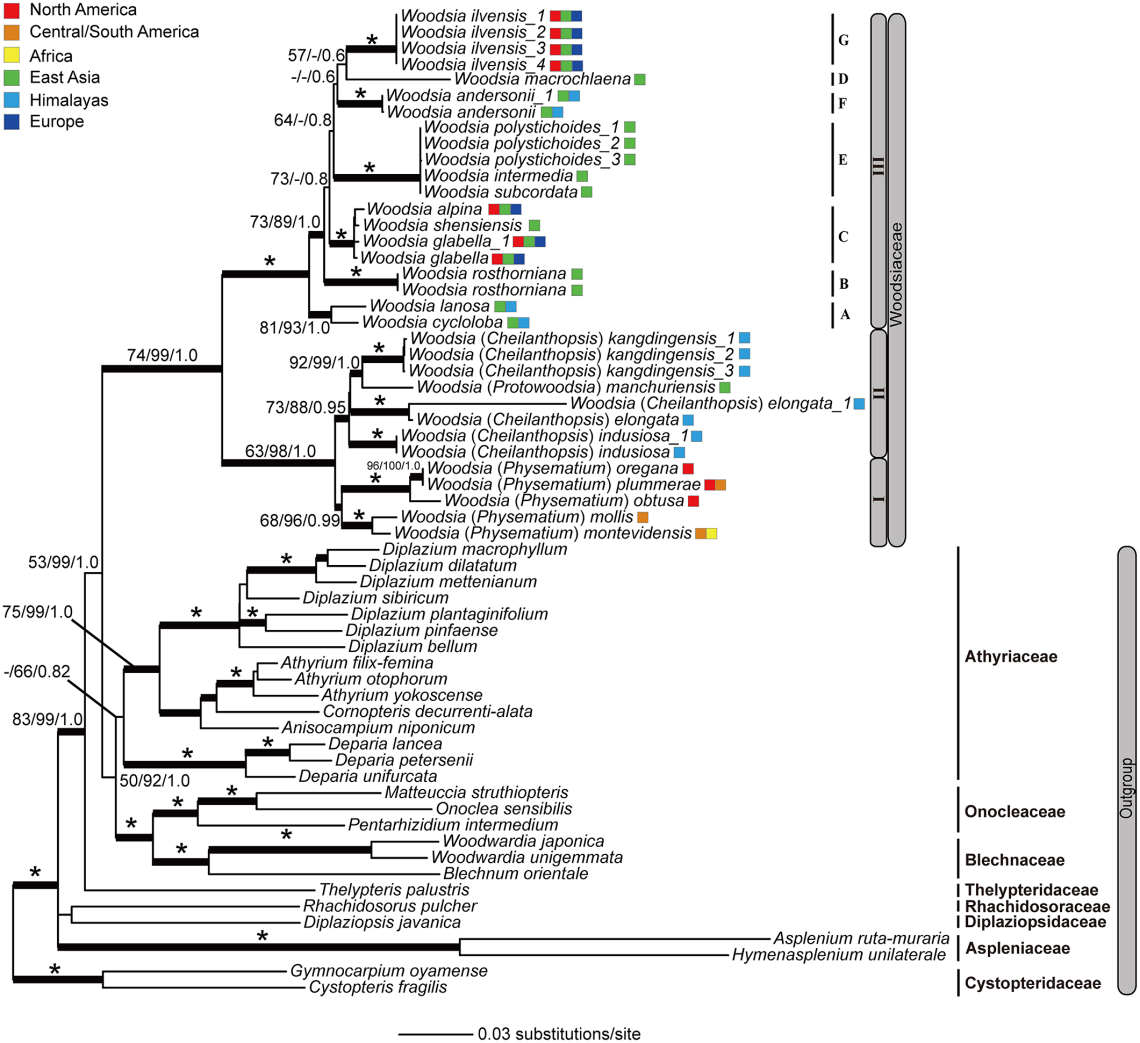
Phylogram of Woodsiaceae obtained from the maximum likelihood analysis of the combined dataset. Numbers on branches are support values [maximum parsimony bootstrap values (BS_MP_)/maximum likelihood bootstrap values (BS_ML_)/Bayesian inference posterior probability values (PP_BI_)]. Bold branches indicate BS_MP_, BS_ML_ ≥ 60% and PP_BI_ ≥ 0.95. Asterisk indicates BS_MP_, BS_ML_ = 100% and PP_BI_ = 1.0. Dash (-) indicates nodes with BS_MP_ or BS_ML_ < 50%. I to III mark the three major clades recognized in Woodsiaceae and A to G represent the seven subclades in clade III. Other lineages in eupolypods II are marked with family names. Colored squares indicate the geographical distributions of each species.

The combined data matrix consists of 4700 aligned base pairs. The characteristics and statistics of individual plastid DNA regions and the combined dataset are presented in Table 2. Three phylogenetic analyses (MP, ML, BI) based on the combined dataset produced highly congruent results (Fig 2). MP analysis yielded 48 most parsimonious trees, with a length of 5672 steps, a consistency index (CI) of 0.46 and a retention index (RI) of 0.72. The ML analysis had a final –ln *L* = 34013.98 (Fig 2). The ESS values for all the estimated parameters in BI analysis were above 200.

Our results show that all Woodsiaceae accessions formed a monophyletic group with strong support (BS_MP_ = 74; BS_ML_ = 99; PP_BI_ = 1.0) (Fig 2). Furthermore, *Woodsia* s.s. and *Physematium* were each recovered to be monophyletic, whereas *Protowoodsia* was nested within *Cheilanthopsis*, and that clade is sister to *Physematium* as typically defined (Fig 2). In total, three well-supported main clades (Fig 2I–2III) and seven robust subclades (Fig 2A–2G) were recovered.

Main clade I (BS_MP_ = 68; BS_ML_ = 93; PP_BI_ = 0.97) corresponds to *Physematium*, a group endemic to America and southern Africa. Main clade II (BS_MP_ = 73; BS_ML_ = 82; PP_BI_ = 1.0) consists of the East Asian and Himalayan species that have been treated as either *Protowoodsia* or *Cheilanthopsis*. Main clade III (BS_MP_, BS_ML_ = 100; PP_BI_ = 1.00) comprises the circumboreal species, most of which occur in Eurasia and northern North America. However, relationships among the seven subclades (A–G) are still unclear due to a poorly resolved backbone (most of the nodes with BS_MP_, BS_ML_ < 70; PP_BI_ < 0.9, see Fig 2).

### Evolution of morphological and cytological characters

**Fig 3:**
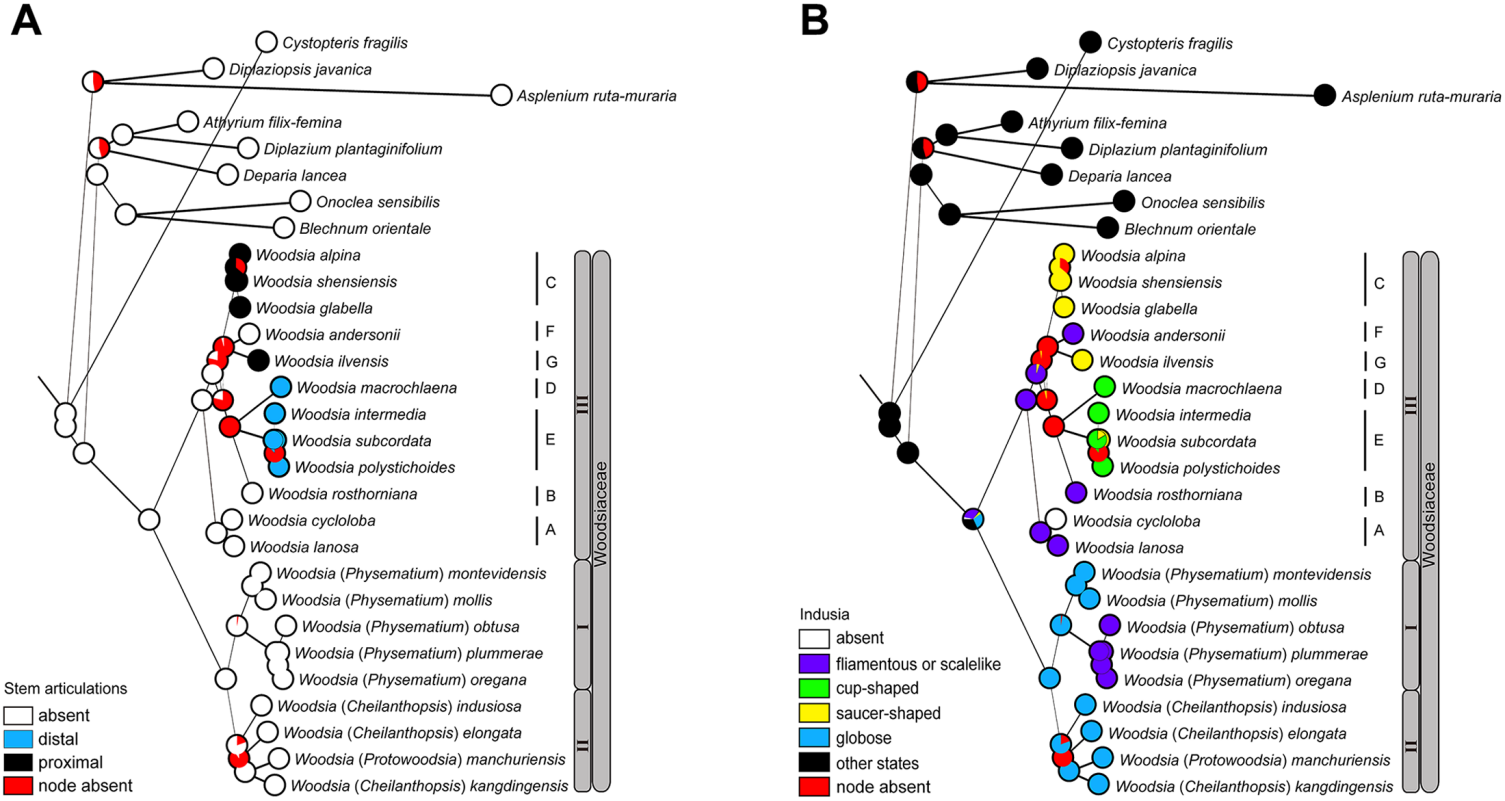
Evolution of selected characters using likelihood method across 1000 BMCMC trees obtained from MrBayes. Pie charts at each node illustrate the likelihood reconstructions and show the proportion of the average likelihood received by each character state as the ancestral character of a given clade. Node absent indicates proportion of nodes with posterior probabilities less than 0.95 across trees. (A) Stem articulations. (B) Indusia. Numbers I to III and letters A to G mark clades and subclades of Woodsiaceae as in Fig 2 and the text.

**Fig 4:**
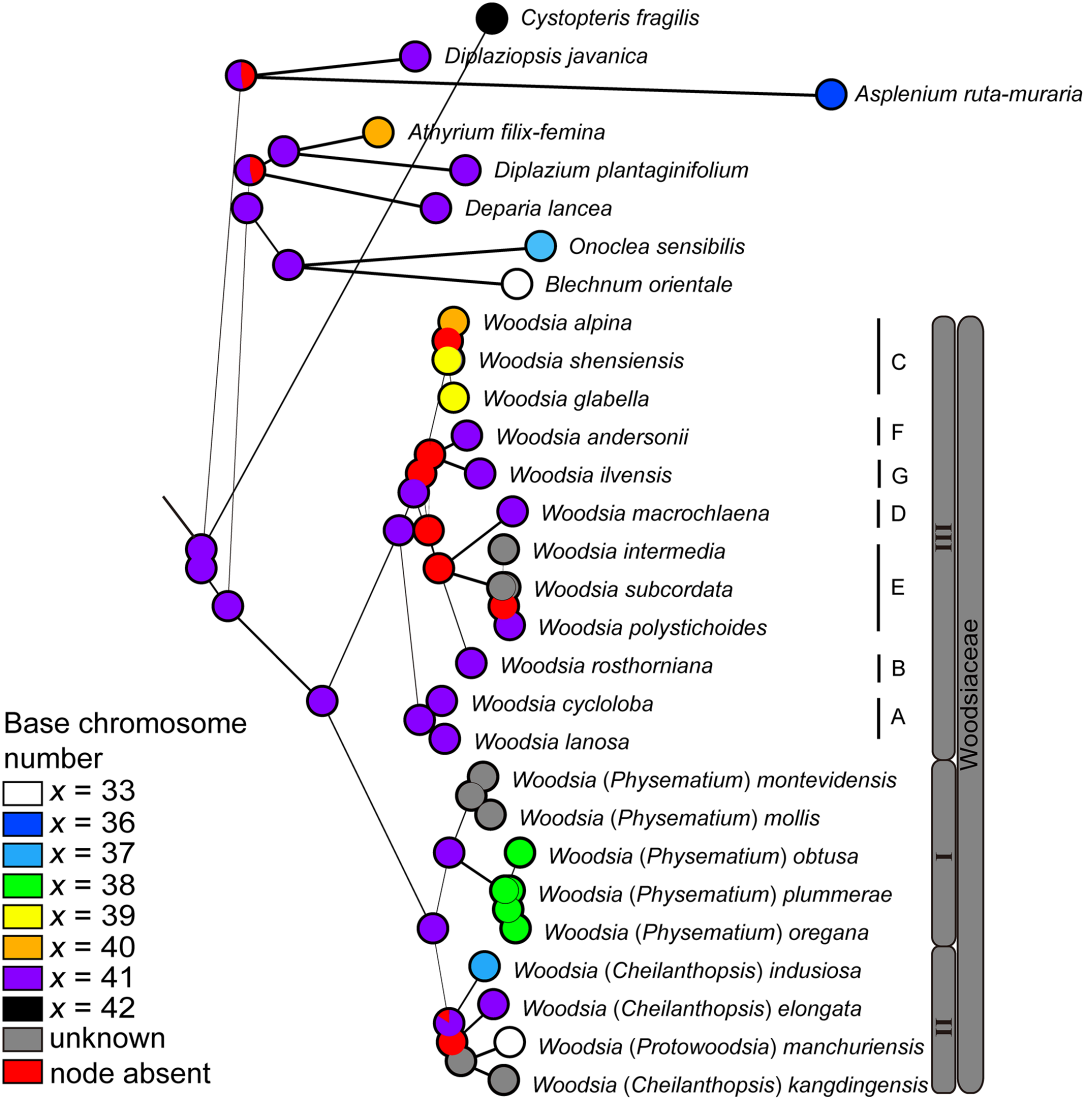
Evolution of chromosome base number using likelihood method across 1000 BMCMC trees obtained from MrBayes. Pie charts at each node illustrate the likelihood reconstructions and show the proportion of the average likelihood received by each character state as the ancestral character of a given clade. Node absent indicates proportion of nodes with posterior probabilities less than 0.95 across trees. Numbers I to III and letters A to G mark clades and subclades of Woodsiaceae as in Fig 2 and the text.

The analyses of the evolution of morphological and cytological characters indicate complex evolutionary patterns in Woodsiaceae and likelihood ancestral character state reconstructions are presented in Figs 3 and 4.

Presence of stem articulations (Fig 3A): “absent” was inferred to be the ancestral state (proportional likelihood [PL]: 1). The state of “distal” and “proximal” are likely derived from “absent”, and all of them experienced parallel evolution.

Shape of indusia (Fig 3B): Reconstructions of the ancestral character states at the crown node of Woodsiaceae are equivocal. Filamentous or scalelike indusia were inferred as the most probable ancestral state in clade III (PL: 1), while globose indusia were reconstructed as the most likely ancestral state for clades I (PL: 0.99) and II (PL: 0.93).

Chromosome base number (Fig 4): chromosome of 41 were inferred as the ancestral state, while other states (*x* = 33, 37, 38, 39, 40) were reconstructed as apomorphic in Woodsiaceae.

## Discussion

### Familial and generic delimitation of Woodsiaceae

Our phylogenetic results show that *Woodsia* including *Cheilanthopsis*, *Protowoodsia* and *Physematium* (BS_MP_ = 74; BS_ML_ = 96; PP_BI_ = 1.00) is a distinct lineage from the athyrioid ferns (*Anisocampium*, *Athyrium*, *Cornopteris*, *Deparia*, and *Diplazium*), in accordance with previous studies of the phylogeny of eupolypod II ferns [2,23,27,28]. Therefore, we confirm the recognition of Woodsiaceae as circumscribed by Christenhusz et al. [1] and Rothfels et al. [2] as separate from Athyriaceae. Woodsiaceae can be delineated on the basis of inferior indusia, stem articulations, and indument mixed with membranaceous scales and raised receptacles [10,29,54]. Furthermore, most species in this family have an epipetric habit [54].

Within Woodsiaceae, *Woodsia* s.s. (as conceived by Ching [8], Wu [5], and Shmakov [11]) and *Physematium* are recovered as monophyletic, whilst *Protowoodsia* is nested within *Cheilanthopsis* (Fig 2). *Cheilanthopsis* is a small genus of about three species restricted to the Himalayan region, and was segregated from *Woodsia* s.s. mainly by the occurrence of false indusia [5,10,31]. However, the false indusia referred to the generic circumscription of *Cheilanthopsis* are only present in one species (*Cheilanthopsis indusiosa*), and are absent in the other two (*C*. *elongata* and *C*. *kangdingensis*). In contrast, *Cheilanthopsis* and *Woodsia* s.s. share some important diagnostic characters, such as septate hairs and granular perispores [4,5,10,54]; there are no character combinations that can be used to unequivocally assign species to *Cheilanthopsis* or *Woodsia*. Therefore, we reject the putative separation of *Cheilanthopsis* from *Woodsia*, a decision supported by previous studies [7,10,31]. The monotypic genus *Protowoodsia* should be incorporated in *Woodsia* as well, given its phylogenetic placement within *Cheilanthopsis* (Fig 2). In addition, the inclusion of *Hymenocystis* (*H*. *fragilis*) into *Woodsia* can be inferred from Larsson [33], in which this genus is sister to *Protowoodsia*.

Although *Physematium* is resolved as monophyletic (Fig 2), morphological characters distinguishing *Physematium* from *Woodsia* (including *Cheilanthopsis* and *Protowoodsia*) appear to be homoplastic according to the evolutionary analyses of morphological characters (see discussion below). For example, filamentous or scalelike indusia characterizing *Physematium obtusum* or *P*. *plummerae* can be also found in some species of *Woodsia*, such as *W*. *andersonii* and *W*. *rosthorniana* (Fig 3B). The recognition of *Physematium* as an independent genus thus is not supported by the present study. Instead, we propose a broadly defined *Woodsia* including *Cheilanthopsis*, *Hymenocystis*, *Protowoodsia* and *Physematium* in the interest of preserving monophyly and taxonomic stability.

### Phylogenetic relationships among clades

Three highly supported “major” clades (I–III) and seven subclades in clade III were recovered by our phylogenetic analyses of *Woodsia* (Fig 2).

Clade I includes species mainly occurring in the New World and extending to southern Africa (also see [33]) (Fig 2). They share characters such as bicolorous stem scales, the indument with capitate hairs, and a chromosome base number of 38 [4,32,54] (Figs 3 and 4). Species in this clade represent a distinct lineage in *Woodsia* [4] that has diversified in the New World.

Species in clade II were previously placed in *Cheilanthopsis* or *Protowoodsia*. The geographic distribution of this clade is centered in Sino-Himalaya regions and extends into northern Asia. The shared morphological characters, such as globose indusia and lack of stem articulations, supports a close relationship among these species [54]. Moreover, short branch lengths deep within the clade (Fig 2) may indicate an ancient rapid diversification [28,55]. The evolutionary history of this clade in the context of geological events and climatic fluctuations resulted from the uplift of Himalayas during the last 60 million years [56,57] would be an interesting topic for study.

Clade III is recovered as the core lineage of *Woodsia*, including the Eurasian and circumboreal species and also the type of this genus (*W*. *ilvensis*). This clade is further split into seven subclades (Fig 2A–2G). However, phylogenetic relationships among these subclades are weakly supported (Fig 2). Moreover, clade III shows greater morphological variability than the other clades (Fig 1A–1D). For instance, most of the species have saucer- or cup-shaped indusia, while the indusia of species from subclades A and B are absent (*W*. *cycloloba*) or composed of curly hairs (*W*. *lanosa*, and *W*. *rosthorniana*). Another example lies in the position of stem articulations: proximal stem articulations mainly occur in subclades C and G, while species from subclades D and E exhibit distal stem articulations. Species identification in this clade is difficult as well. *Woodsia polystichoides*, *W*. *macrochlaena*, *W*. *subcordata*, and *W*. *intermedia* can be easily confused due to continuous variation in leaf shape, indument, and costa scales [6,58]. Unfortunately, the phylogenetic relationships of these three species are poorly resolved: there is no support between *W*. *macrochlaena* (subclade D) and subclade E and little genetic differentiation between *W*. *polystichoides* and *W*. *intermedia*. Clade III is in need of further taxonomic and phylogenetic investigation.

Although our study comprises 21 out of 35 species, our samples include representatives of all groups, reflecting the morphological divergence of Woodsiaceae in Eurasia, North/South America and Africa [4,19,35]. For example, the morphological representatives *W*. *ilvensis*, *W*. (*Cheilanthopsis*) *elongata*, *W*. (*Protowoodsia*) *manchuriensis*, and *W*. (*Physematium*) *montevidensis* are here included. An increased sampling will be much helpful to test hypotheses concerning local diversity or aim to reconstruct the colonization of the New World in greater detail. However, it will not substantially alter the main phylogenetic topology recovered in our study [33].

### Inferred evolution of *Woodsia* morphology

Our ancestral character reconstruction reveals stem articulations are restricted in some subclades within clade III (Fig 3A). This result suggests that proximal stem articulations and distal stem articulations can be employed as one of the diagnostic characters for these lineages, such as subclades C, D, E, and G.

As for the other selected morphological character, the ancestral state of indusia is ambiguous for Woodsiaceae due to an equivocal reconstruction (Fig 3B). Our analyses reveal that the shape of indusia, on which previous treatments were based (e.g., [6,7,10,30]), exhibits a high level of homoplasy (Fig 3B). For example, filamentous or scalelike indusia are inferred to have independently evolved in clades I and III, respectively (Fig 3B). Therefore, many of the genera or infrageneric ranks of Woodsiaceae should be rejected or redefined in our context of combined investigation of morphology and phylogenetic framework (see taxonomic treatments).

Our likelihood reconstruction show *x* = 41 to be the most likely ancestral state of the chromosome number within *Woodsia* (Fig 4), which is consistent with a previous study [54]. This inference is also supported by the fact that most species of Woodsiaceae possess a chromosome base number as 41 [32,51]. Records of 38, 39 and 40 were derived from 41 secondarily. This result provides evidence for the evolution pathway of chromosome number from 41 to 38, 39 and 40 caused by aneuploid reduction [23,59], and similar pattern also has been found in other fern groups, such as *Lepisorus* in Polypodiaceae [60]. The evolutionary pattern of chromosome base number in clade II is more complicated than in the other clades (Fig 4). No evidence was found to support the hypotheses that *x* = 33 is an ancestral state in Woodsiaceae [32]. Furthermore, our analysis offers some evidence against the hypothesis that *x* = 37 (*W*. *indusiosa*) originated from the hybridization of *x* = 41 (*W*. *elongata*) and *x* = 33 (*W*. *manchuriensis*) taxa [32]. According to this hypothesis, a clustering of *W*. *indusiosa* with its maternal species (*W*. *elongata* or *W*. *manchuriensis*) should be recovered, whereas these species are diverged from each other in our phylogeny (Figs 2 and 4).

## Taxonomic Treatments

Given the phylogenetic framework and patterns of morphological evidence revealed in our study, we propose a broadly defined *Woodsia* within Woodsiaceae. The alternative would be to redefine the segregates according to the phylogenetic framework. However, doing so would require the introduction of smaller genera with poor morphological cohesiveness. Here, we attempt to propose a subgeneric classification of *Woodsia* to manage the morphological diversity of this broadly defined genus, to maintain the taxonomic stability, and also to consider the DNA sequence-based phylogeny, morphological variation and geographic distribution. Clades I and II recovered in our phylogenetic analyses are congruent with subgenera *Physematium* and *Cheilanthopsis*, respectively, while clade III, in which the type species (*W*. *ilvensis*) for *Woodsia* is nested, corresponds to subgenus *Woodsia*.

***Woodsia*** R. Br. in Prodr. Fl. Nov. Holland: 158, Obs. 4. 1810;

**Type.** *Woodsia ilvensis* (L.) R. Br.

**Description.** Plants small to medium sized; usually lithophytic. Rhizomes short, erect, decumbent, or ascending, covered with scales. Fronds clustered, monomorphic, deciduous or sometimes evergreen; stems usually covered with scales and septate hairs, articulate or not; lamina 1-pinnate to bipinnatifid, elliptic-lanceolate to narrowly lanceolate, frequently covered with articulate (septate) hairs, sometimes with glandular hairs or capitate glands. Veins free, pinnate, usually ending in enlarged hydathodes. Sori orbicular; indusia inferior, globose (subg. *Physematium* & subg. *Cheilanthopsis*) or saucer-shaped to cup-shaped, margin long ciliate, or indusia absent, degenerated into curly hairs (subg. *Woodsia*), or sometimes also covered with false indusia, i.e., reflexed leaf margins (e.g., *W*. *indusiosa*). Spores ellipsoid or somewhat spherical, monolete, wall surface folded, cristate, tuberculate, or echinate.

### Synonyms

***Physematium*** Kaulf. in Flora 12: 341. 1829;

**Type.** *Physematium molle* Kaulf., Flora 12: 341. 1829.

***Hymenocystis*** C.A. Mey. in Verz. Pfl. Casp. Meer. (C.A. von Meyer).: 229. 1831

**Type.** *Hymenocystis fragilis* (Trev.) Askerov (= *H*. *caucasica* C.A. Mey.), Izv. Akad. Nauk Azerbajdzansk. SSR, Ser. Biol. Med. Nauk 3: 52. 1986.

***Cheilanthopsis*** Hieron. in Notizbl. Bot. Gart. Berlin-Dahlem 7: 406. 1920.

**Type.** *Cheilanthopsis indusiosa* (Christ) Ching (= *C*. *straminea* (Brause) Hieron.), Sinensia 3: 154. 1932.

***Protowoodsia*** Ching in Sunyatsenia 5: 245. 1940

**Type.** *Protowoodsia manchuriensis* (Hook.) Ching, Sunyatsenia 5: 245.

### Key to the subgenera of *Woodsia*

1. Stem articulations present; leaves with needle-like articulate hairs; indusia non-globose (saucer- to cup-shaped, or reduced into uniseriate, septate hairs) or exindusiate; *x* = 39, 41; plants distributed in Northern Hemisphere, sometimes circumboreal………subg. ***Woodsia***
1. Stem articulations absent; indusia mostly globose or with multiseriate filamentous or scale-like segments………………………………………………………………………2
2. Rhizome scales bicolorous; leaves with capitate hairs; sori with multiseriate filamentous or scale-like segments (indusia), or sometimes globose, but never with false indusia; *x* = 38; plants mainly distributed in North/South America, extending to southern Africa………………………………………………………………………………subg. ***Physematium***
2. Rhizome scales concolorous; leaves with sparse or sometimes cylindric articulate hairs; sori with globose indusia, occasionally with false indusia; *x* = 33, 37, 41; plants mainly restricted to the Sino-Himalaya regions……………………………subg. ***Cheilanthopsis***

Subgenus 1. ***Woodsia*** subg. ***Woodsia***

**Type.** *Woodsia ilvensis* (L.) R. Br.

**Description.** Plants small. Rhizomes short, erect or ascending, rarely decumbent, scaly; scales concolorous. Fronds clustered; stems articulate at various levels (i.e., lower part near base, or continuous to the top at upper part); lamina 1-pinnate or 1-pinnate-pinnatifid, lanceolate, usually with needle-like articulate hairs or scales, rarely glabrous, base gradually tapering; pinnules margin undulate or entire. Sori small, orbicular; indusia inferior, various, but never globose, usually saucer- to cup-shaped, or reduced into curly, uniseriate septate hairs, sometimes exindusiate. Spores ellipsoid, monolete, perispore folded with granular, echinate or tuberculate. Chromosome number: *x* = 39, 41.

**Species and Distribution.** About 19 species in Eurasia and circumboreal region, e.g., *W*. *alpina* (Bolton) Gray, *W*. *andersonii* (Bedd.) Christ, *W*. *cycloloba* (Bedd.) Christ, *W*. *glabella* R. Br. ex Richardson, *W*. *ilvensis* (L.) R. Br., *W*. *lanosa* Hook., *W*. *polystichoides* D.C. Eaton, *W*. *rosthorniana* Diels etc.

Subgenus 2. ***Woodsia*** subg. ***Physematium*** (Kaulf.) Hook. emend. X.C. Zhang & R. Wei

**Type.** *Woodsia mollis* (Kaulf.) J. Sm.

**Basionym.** *Physematium* Kaulf. in Flora 12: 341. 1829

**Synonym.** *Woodsia* subg. *Perrinia* Hook. in Sp. Fil. 1: 62. 1846. **syn. nov.**

**Description.** Plants medium sized. Rhizomes erect, short, apex densely scaly; scale bicolorous. Fronds clustered; stems not articulate; lamina 1-pinnate pinnatifid, lanceolate, gradually reduced distally to pinnatifid apex, usually with capitate hairs; pinnae not articulate to rachis; pinnules margin dentate or occasionally entire (*W*. *mollis*). Sori small, orbicular; indusia inferior, dissected into several or numerous multiseriate filamentous or scale-like segments, or sometimes globose. Spores ellipsoid, monolete, perispores cristate, rarely rugose. Chromosome number: *x* = 38.

**Species and Distribution.** About 10 to 15 species in America, also extending to southern Africa, e.g., *W*. *angolensis* Schelpe, *W*. *mexicana* R. Br., *W*. *mollis* (Kaulf.) J. Sm., *W*. *montevidensis* (Spreng.) Hieron., *W*. *obtusa* Torr., *W*. *oregana* D.C. Eaton, *W*. *plummerae* Lemmon, *W*. *scopulina* D.C. Eaton, etc.

**Note.** The subgenus defined here is not identical to the concept of Hooker [61]. For example, *W*. *elongata* is excluded, while subg. *Perrinia* Hook. described in Hooker [61] is included.

Subgenus 3. ***Woodsia*** subg. ***Cheilanthopsis*** (Hieron.) X.C. Zhang & R. Wei, **comb. & stat. nov.**

**Type.** *Woodsia indusiosa* Christ

**Basionym.** *Cheilanthopsis* Hieron. in Notizbl. Bot. Gart. Berlin-Dahlem 7: 406. 1920

**Description.** Plants small to medium sized. Rhizomes erect, short, apex densely scaly; scale concolorous. Fronds clustered or subclustered, usually diverging; stems not articulate; lamina 1-pinnate pinnatifid, lanceolate, gradually reduced distally to pinnatifid apex, hairs spares or sometimes with cylindric articulate hairs; pinnae sometimes articulate to rachis; pinnules margin undulate (*W*. *elongata*, *W*. *indusiosa* and *W*. *manchuriensis*) or entire (*W*. *kangdingensis* and *W*. *fragilis*). Veins free, pinnate, not reaching laminar margin. Sori small, orbicular; indusia inferior, globose, completely wrapping sporangia, tearing apically at maturity, or sometimes covered with false indusia. Spores ellipsoid, monolete, perispores corrugate. Chromosome number: *x* = 33, 37, 41.

**Species and Distribution.** Five species mainly in the Pan-Himalaya, also extending to northern Asia, Japan and Far East: *W*. *elongata* Hook., *W*. *fragilis* Liebm., *W*. *indusiosa* Christ, *W*. *kangdingensis* H.S. Kung, Li Bing Zhang & X.S. Guo, *W*. *manchuriensis* Hook.

## Supporting Information

S1 Table Species names and GenBank accession numbers of DNA sequences used in this study. **–**: indicates data not available. *: means newly generated sequences in this study.
(DOC)

S2 Table Matrix of morphological character states used to reconstruct the evolution of these characters. Morphological characters: (1) stem articulations: 0 = absent; 1 = distal; 2 = proximal; (2) indusia: 0 = absent; 1 = curly hairs; 2 = globose; 3 = cup-shaped; 4 = saucer-shaped; 5 = other states; (3) basic chromosome number: 0: x = 33; 1: x = 36; 2: x = 37; 3: x = 38; 4: x = 39; 5: x = 40; 6: x = 41; 7: x = 42. Missing data were codes as ‘‘?”.
(DOC)
