# Supplementary material for: Molecular Phylogeny of the Cliff Ferns (Woodsiaceae: Polypodiales) with a Proposed Infrageneric Classification
Source: PLoS One. 2015 Sep 8;10(9):e0136318. doi: 10.1371/journal.pone.0136318 (PMC4562699; doi:10.1371/journal.pone.0136318)
Supplement: S1 Table — –: indicates data not available. *: means newly generated sequences in this study. (DOC) [file pone.0136318.s001.doc]

**Table S1.** Species names and GenBank accession numbers of DNA sequences used in this study. –: indicates data not available. *: means newly generated sequences in this study.

| **Species** | **Locality** | **Voucher (herbarium)** | **Genbank accession numbers** | | | | **References** |
| --- | --- | --- | --- | --- | --- | --- | --- |
|  |  |  | ***atp*A** | ***mat*K** | ***rbc*L** | ***trn*L-F** |  |
| **Ingroup taxa** |  |  |  |  |  |  |  |
| *Woodsia alpina* (Boltan) Gray | Sichuan, China | Kham Exp 10-247 (PE) | KP226709* | KP226734* | KP226759* | KP226784* | This study |
| *Woodsia andersonii* (Bedd.) Christ | Sichuan, China | X.-C. Zhang 5797 (PE) | KP226710* | KP226735* | KP226760* | KP226785* | This study |
|  | Sichuan, China | Kham Exp 10-246 (PE) | KP226711* | KP226736* | KP226761* | KP226786* | This study |
| *Woodsia cycloloba* Hand.-Mazz. | Xizang, China | X.-C. Zhang 5114 (PE) | KP226712* | KP226737* | KP226762* | KP226787* | This study |
| *Woodsia* (*Cheilanthopsis*) *elongata* Hook. | China | S.G. LU/JU33 | JN168053 | – | JN168007 | – | [1] |
|  | Maharigaon, Nepal | Polunin, Sykes & Williams (UPS) | JF832103 | – | JF832060 | – | [2] |
| *Woodsia glabella* R. Br. ex Richards. | Heilongjiang, China | X.-C. Zhang 6874 (PE) | KP226713* | KP226738* | KP226763* | KP226788* | This study |
|  | Xinjiang, China | X.-C. Zhang 4336 (PE) | KP226714* | KP226739* | KP226764* | KP226789* | This study |
| *Woodsia ilvensis* (L.) R. Br. | Russia | – | KP226715* | KP226740* | KP226765* | KP226790* | This study |
|  | Heilongjiang, China | X.-C. Zhang 6878 (PE) | KP226716* | KP226741* | KP226766* | KP226791* | This study |
|  | Hubei, China | R. Wei et al. s.n. (PE) | KP226717* | KP226742* | KP226767* | KP226792* | This study |
|  | Beijing, China | R. Wei WR0175 (PE) | KP226718* | KP226743* | KP226768* | KP226793* | This study |
| *Woodsia* (*Cheilanthopsis*) *indusiosa* Christ | Yunnan, China | X.-C. Zhang 6464 (PE) | KP226703* | KP226728* | KP226753* | KP226778* | This study |
|  | Sichuan, China | X.-C. Zhang 2481 (PE) | KP226704* | KP226729* | KP226754* | KP226779* | This study |
| *Woodsia intermedia* Tagawa | Japan | TNS:743747 | – | – | AB574999 | – | [3] |
| *Woodsia* (*Cheilanthopsis*) *kangdingensis* H.S. Kung, Libin Zhang & X.S. Guo | Sichuan, China | X.-C. Zhang 5808 (PE) | KP226705* | KP226730* | KP226755* | KP226780* | This study |
|  | Sichuan, China | X.-C. Zhang 5839 (PE) | KP226706* | KP226731* | KP226756* | KP226781* | This study |
|  | Sichuan, China | X.-C. Zhang 5852 (PE) | KP226707* | KP226732* | KP226757* | KP226782* | This study |
| *Woodsia lanosa* Hook. | Yunnan, China | Li Z-Y 497 (PE) | KP226719* | KP226744* | KP226769* | KP226794* | This study |
| *Woodsia macrochlaena* Mett. ex Kuhn | Heilongjiang, China | X.-C. Zhang 6873 (PE) | KP226720* | KP226745* | KP226770* | KP226795* | This study |
| *Woodsia* (*Protowoodsia*) *manchuriensis* Hook. | South Korea | X.-C. Zhang 2398 (PE) | KP226708* | KP226733* | KP226758* | KP226783* | This study |
| *Woodsia* (*Physematium*) *mollis* (Kaulf.) J. Sm. | Hidalgo, Mexico | Larsson 103 (UPS) | JF832148 | JF832294 | JF832087 | – | [2] |
| *Woodsia* (*Physematium*) *montevidensis* (Spreng.) Hieron. | Bolivia | M. Kessler s.n. (Z) | KP226721* | KP226746* | KP226771* | KP226796* | This study |
| *Woodsia* (*Physematium*) *obtusa* Torr. | In cultivation | Schuettpelz 328 (DUKE) | EF463923 | – | EF463319 | – | [4] |
| *Woodsia* (*Physematium*) *oregana* D.C. Eaton | Manitoulin Island, Canada | OAC 96880 | KF186555 | – | KF186523 | – | – |
| *Woodsia* (*Physematium*) *plummerae* Lemmon | Arizona, America | Schuettpelz 1235A (DUKE) | JF832149 | JF832295 | JF832088 | – | [2] |
| *Woodsia polystichoides* D.C. Eaton | Hubei, China | R. Wei WR0159 (PE) | KP226722* | KP226747* | KP226772* | KP226797* | This study |
|  | Hubei, China | X.-C. Zhang 3354 (PE) | KP226723* | KP226748* | KP226773* | KP226798* | This study |
|  | Henan, China | Wu C ch0801001 (PE) | KP226724* | KP226749* | KP226774* | KP226799* | This study |
| *Woodsia rosthorniana* Diels | Sichuan, China | Kham Exp 10-426 (PE) | KP226725* | KP226750* | KP226775* | KP226800* | This study |
|  | Sichuan, China | X.-C. Zhang 6495 (PE) | KP226726* | KP226751* | KP226776* | KP226801* | This study |
| *Woodsia shensiensis* Ching | Hubei, China | X.-C. Zhang 3438 (PE) | KP226727* | KP226752* | KP226777* | KP226802* | This study |
| *Woodsia subcordata* Turcz. | Japan | TNS:776980 | – | – | AB575003 | – | [3] |
| **Outgroup taxa** | – | – |  |  |  |  |  |
| *Anisocampium niponicum* (Mett.) Y.C. Liu, W.L. Chiou & M. Kato | – | – | JF832097 | JF832257 | JF832057 | AF515256 | [2,5] |
| *Asplenium ruta-muraria* L. | – | – | EF463608 | JF832253 | AF525273 | HQ676517 | [2,4,6,7] |
| *Athyrium filix-femina* (L.) Roth | – | – | JF832096 | JF303941 | JF832056 | EU329076 | [2,8] |
| *Athyrium otophorum* (Miq.) Koidz. | – | – | JF832098 | JF832258 | EF463305 | AF515236 | [2,4,5] |
| *Athyrium yokoscense* (Franch. & Sav.) Christ | – | – | EF463905 | – | JN168078 | EU329098 | [1,4,8] |
| *Blechnum orientale* L. | – | – | KC254194 | KC254115 | KC254350 | KC254424 | [9] |
| *Cornopteris decurrenti-alata* (Hook.) Nakai | – | – | JF832104 | JF832263 | JF832061 | EU329106 | [2,8] |
| *Cystopteris fragilis* (L.) Bernh. | – | – | JF832108 | JF832062 | AF425148 | HQ676522 | [2,7,10] |
| *Deparia lancea* (Thunb.) Fraser-Jenk. | – | – | JF832109 | JF303940 | EF463306 | AF515238 | [2,4,5] |
| *Deparia petersenii* (Kunze) M. Kato | – | – | JF832110 | JN673852 | JF832065 | JN673894 | [1,2] |
| *Deparia unifurcata* (Baker) M. Kato | – | – | JF832111 | JF832271 | EF463307 | AF515235 | [2,4,5] |
| *Diplaziopsis javanica* (Blume) C. Chr. | – | – | JF832114 | JF303928 | HQ380212 | KC254503 | [2,9,11] |
| *Diplazium bellum* (C.B. Clarke) Bir | – | – | KC254200 | KC254121 | KC254356 | KC254428 | [9] |
| *Diplazium dilatatum* Blume | – | – | KC254267 | KC254188 | KC254418 | KC254497 | [9] |
| *Diplazium macrophyllum* Desv. | – | – | KC254247 | KC254167 | KC254400 | KC254475 | [9] |
| *Diplazium mettenianum* (Miq.) C. Chr. | – | – | KC254212 | KC254133 | KC254368 | KC254440 | [9] |
| *Diplazium pinfaense* Ching | – | – | KC254202 | KC254123 | KC254358 | KC254430 | [9] |
| *Diplazium plantaginifolium* (L.) Urb. | – | – | KC254205 | KC254126 | KC254361 | KC254433 | [9] |
| *Diplazium sibiricum* var. *sibiricum* (Turcz. ex Kunze) Sa. Kurata | – | – | KC254208 | KC254129 | KC254364 | KC254436 | [9] |
| *Gymnocarpium oyamense* Ching | – | – | JF832121 | JF832278 | JF832069 | AF515248 | [2,5] |
| *Hymenasplenium unilaterale* (Lam.) Hayata | – | – | JF832127 | EF452020 | EF452140 | AF525232 | [2,4,5] |
| *Matteuccia* (*Onoclea*) *struthiopteris* (L.) Tod. | – | – | KC254197 | KC254118 | KC254353 | KC254425 | [9] |
| *Onoclea sensibilis* L. | – | – | KC254196 | KC254117 | KC254352 | – | [9] |
| *Pentarhizidium* (*Onoclea*) *intermedium* (C. Chr.) Hayata | – | – | KC254198 | KC254119 | KC254354 | KC254426 | [9] |
| *Rhachidosorus pulcher* (Tagawa) Ching | – | – | JF303998 | JF303962 | JF303971 | – | [12] |
| *Thelypteris palustris* Schott | – | – | JF832146 | JF832292 | JF832085 | HQ676538 | [2,7] |
| *Woodwardia unigemmata* (Makino) Nakai | – | – | KC254195 | KC254116 | KC254350 | – | [9] |
| *Woodwardia japonica* (L. f.) Sm. | – | – | JF304006 | JF303937 | AB040600 | DQ683432 | [12,13] |

**References**

1. Li CX, Lu SG, Sun XY, Yang Q (2011) Phylogenetic positions of the enigmatic asiatic fern genera *Diplaziopsis* and *Rhachidosorus* from analyses of four plastid genes. American Fern Journal 101: 142–155.
2. Rothfels CJ, Larsson A, Kuo LY, Korall P, Chiou WL, Pryer KM (2012) Overcoming deep roots, fast rates, and short internodes to resolve the ancient rapid radiation of eupolypod II ferns. Systematic Biology 61: 490–509.
3. Ebihara A, Nitta JH, Ito M (2010) Molecular species identification with rich floristic sampling: DNA barcoding the pteridophyte flora of Japan. PLoS ONE 5: e15136.
4. Schuettpelz E, Pryer KM (2007) Fern phylogeny inferred from 400 leptosporangiate species and three plastid genes. Taxon 56: 1037–1050.
5. Wang ML, Chen ZD, Zhang XC, Lu SG, Zhao, GF (2003) Phylogeny of the Athyriaceae: evidence from chloroplast *trn*L-F region sequences. Acta Phytotaxonomica Sinica 41: 416–426.
6. Pinter I, Bakker F, Barrett J, Cox C, Gibby M, Henderson S, Morgan-Richards M, Rumsey F, Russell S, Trewick S, Schneider H, Vogel,J (2002) Phylogenetic and biosystematic relationships in four highly disjunct polyploid complexes in the subgenera *Ceterach* and *Phyllitis* in *Asplenium* (Aspleniaceae). Organisms Diversity and Evolution 2: 299−311.
7. de Groot GA, During HJ, Maas JW, Schneider H, Vogel JC, Erkens RH (2011) Use of *rbc*L and *trn*L-F as a two-locus DNA barcode for identification of NW-European ferns: an ecological perspective. PLoS ONE 6: e16371.
8. Adjie B, Takamiya M, Ohta M, Ohsawa TA, Watano Y (2008) Molecular phylogeny of the lady fern genus *Athyrium* in Japan based on chloroplast *rbc*L and *trn*L-*trn*F sequences. Acta Phytotaxonomica et Geobotanica 9: 79−95.
9. Wei R, Schneider H, Zhang XC (2013) Toward a new circumscription of the twinsorus-fern genus *Diplazium* (Athyriaceae): a molecular phylogeny with morphological implications and infrageneric taxonomy. Taxon 62: 441–457.
10. Smith AR, Cranfill RB (2002) Intrafamilial relationships of the thelypteroid ferns (Thelypteridaceae). American Fern Journal 92: 131–149.
11. Wei R, Zhang XC (2010) Phylogeny of *Diplaziopsis* and *Homalosorus* based on two chloroplast DNA sequences: *rbc*L and *rps*4+*rps*4-*trn*S IGS. Acta Botanica Yunnanica S17: 46–54.
12. Kuo LY, Li FW, Chiou WL, Wang CN (2011) First insights into fern matK phylogeny. Molecular Phylogenetics and Evolution 59: 556–566.
13. Shepherd LD, Perrie LR, Parris BS, Brownsey PJ (2007) A molecular phylogeny for the New Zealand Blechnaceae ferns from analyses of chloroplast *trn*L-*trn*F DNA sequences. New Zealand Journal of Botany 45: 67–80.
