# Supplementary material for: Molecular Phylogeny of the Cliff Ferns (Woodsiaceae: Polypodiales) with a Proposed Infrageneric Classification
Source: PLoS One. 2015 Sep 8;10(9):e0136318. doi: 10.1371/journal.pone.0136318 (PMC4562699; doi:10.1371/journal.pone.0136318)
Supplement: S2 Table — Morphological characters: (1) stem articulations: 0 = absent; 1 = distal; 2 = proximal; (2) indusia: 0 = absent; 1 = curly hairs; 2 = globose; 3 = cup-shaped; 4 = saucer-shaped; 5 = other states; (3) basic chromosome number: 0: x = 33; 1: x = 36; 2: x = 37; 3: x = 38; 4: x = 39; 5: x = 40; 6: x = 41; 7: x = 42. Missing data were codes as ‘‘?”. (DOC) [file pone.0136318.s002.doc]

**Table S2.** Matrix of morphological character states used to reconstruct the evolution of these characters. Morphological characters: (1) stem articulations: 0 = absent; 1 = distal; 2 = proximal; (2) indusia: 0 = absent; 1 = curly hairs; 2 = globose; 3 = cup-shaped; 4 = saucer-shaped; 5 = other states; (3) basic chromosome number: 0: *x* = 33; 1: *x* = 36; 2: *x* = 37; 3: *x* = 38; 4: *x* = 39; 5: *x* = 40; 6: *x* = 41; 7: *x* = 42. Missing data were codes as ‘‘?”.

| **Species** | **Characters** | | |
| --- | --- | --- | --- |
|  | **Stem articulations** | **Indusia** | **Chromosome base number** |
| *Cheilanthopsis elongata* | 0 | 2 | 6 |
| *Cheilanthopsis indusiosa* | 0 | 2 | 2 |
| *Cheilanthopsis kangdingensis* | 0 | 2 | ? |
| *Protowoodsia manchuriensis* | 0 | 2 | 0 |
| *Woodsia alpina* | 2 | 4 | 5 |
| *Woodsia andersonii* | 0 | 1 | 6 |
| *Woodsia cycloloba* | 0 | 0 | 6 |
| *Woodsia glabella* | 2 | 4 | 4 |
| *Woodsia ilvensis* | 2 | 4 | 6 |
| *Woodsia intermedia* | 1 | 3 | ? |
| *Woodsia macrochlaena* | 1 | 3 | 6 |
| *Woodsia lanosa* | 0 | 1 | 6 |
| *Woodsia mollis* | 0 | 2 | ? |
| *Woodsia montevidensis* | 0 | 2 | ? |
| *Woodsia obtusa* | 0 | 2 | 3 |
| *Woodsia oregana* | 0 | 2 | 3 |
| *Woodsia plummerae* | 0 | 2 | 3 |
| *Woodsia polystichoides* | 1 | 3 | 6 |
| *Woodsia rosthorniana* | 0 | 1 | 6 |
| *Woodsia shensiensis* | 2 | 4 | ? |
| *Woodsia subcordata* | 1 | 4 | ? |
| *Athyrium filix-femina* | 0 | 5 | 5 |
| *Asplenium ruta-muraria* | 0 | 5 | 1 |
| *Blechnum orientale* | 0 | 5 | 0 |
| *Cystopteris fragilis* | 0 | 5 | 7 |
| *Deparia lancea* | 0 | 5 | 6 |
| *Diplaziopsis javanica* | 0 | 5 | 6 |
| *Diplazium plantaginifolium* | 0 | 5 | 6 |
| *Onoclea sensibilis* | 0 | 5 | 2 |
